# Serum Se, Ni, and As are associated with HPV infection and CIN2+ among Uyghur women in rural China

**DOI:** 10.1186/s12885-018-4734-6

**Published:** 2018-09-26

**Authors:** Guzhalinuer Abulizi, Yuan-Yuan Zhang, Patiman Mijiti, Hua Li, Guzhalinuer Abuduxikuer, Jing Cai, Zhi-Hong Dong, Gulimire Naizhaer, Xiu-Wei Yang, Miherinisha Maimaiti, Ling-Lu, Guligeina Abudurexiti, Gulixian Tuerxun, Kailibinuer Aierken, Yu-Jie Jiang, Li-Li, Ming-Yue Zhu, Lu Zhang, Tangnuer Abulimiti

**Affiliations:** 1 5th Department of Gynecology, Affiliated Tumor Hospital of Xinjiang Medical University, No. 789 Suzhou East Road, Urumqi City, 830054 Xinjiang Uyghur Autonomous Region China; 2 Gynecological Clinic, Affiliated Tumor Hospital of Xinjiang Medical University, Urumqi City, 830054 Xinjiang Uyghur Autonomous Region China; 3 3rd Department of Gynecologic, Affiliated Tumor Hospital of Xinjiang Medical University, Urumqi City, 830054 Xinjiang Uyghur Autonomous Region China; 4 2nd Department of Gynecology, First People’s Hospital of Xinjiang Kashgar, No.66, Airport Street, Kashgar, 844000 Xinjiang Uyghur Autonomous Region China; 5 Department of Gynecology, People’s Hospital of Xinjiang Uyghur Autonomous Region, 91 Tian Chi Road, Urumqi, 830001 Xinjiang Uyghur Autonomous Region China

**Keywords:** Cervical lesions, Trace elements, HPV, Uyghur

## Abstract

**Background:**

Cervical cancer incidence and mortality is high in Uyghur ethnics. Their life style and dietary habit were different from other ethnics living together. Study on the role of trace elements in HPV infection and cervical lesion of Uyghur minority is needed for future intervention and prevention work.

**Methods:**

In total, 833 Uyghur women were randomly selected from the screening site and hospital. The concentrations of the trace elements As, Fe, Cd, Ni, Cu, Zn, Mn, and Se were determined by atomic absorption spectrophotometry and inductively coupled plasma atomic emission spectroscopy. Univariate analysis was performed with chi-squared test between the HPV-positive and HPV-negative groups and between the case group and the control group. Multivariate analysis was performed with logistic regression.

**Results:**

An As concentration ≥ 0.02 mg/kg was a risk factor for HPV infection (OR > 1, *P* < 0.05), and Ni concentration ≥ 0.1232 mg/kg and Se concentration ≥ 0.02 mg/kg were protective factors (OR < 1, *P* < 0.05). Concentrations of Fe ≥ 6.9153 mmol/L and As ≥0.02 mg/kg were risk factors for CIN2+ (OR > 1, *P* < 0.05), and concentrations of Ni ≥0.0965 mg/kg and Se ≥0.02 mg/kg were protective factors (OR < 1, *P* < 0.05).

**Conclusions:**

Low serum concentrations of Se and Ni and a high serum concentration of As might be related to HPV infection and CIN2+ in Uyghur women in rural China.

## Background

Cervical cancer is one of the most prevalent cancers worldwide, and the most recent compilation of data indicates that an estimated 470,000 new cervical cancer cases occur annually among women worldwide. An estimated 65,105 new cases developed, and 31,998 deaths occurred in Chinese women in 2015 [1]. Evidences from numerous studies have shown that human papillomavirus (HPV) infection is a major risk factor for cervical dysplasia and is present in 99.7% of cervical carcinomas [2]. While 80% of women will be infected with HPV at some point during their lifetime [3], the majority of women with HPV infection do not develop cervical neoplasia, which suggests that other cofactors are involved in cervical cancer pathogenesis.

Trace elements are essential for the normal function of different metabolic processes in the body. Studies have shown that Se, Fe, As, Cu, Ni, Zn, Cd and Mn are essential trace elements that play an important role in malignant tumor incidence and progression [4, 5].

A variety of studies have reported an increase in the levels of Cu in serum of patients with a malignancy. This increase has been found in carcinomas such as gastric, mammary, cervical, bladder and bronchial carcinoma [6–10]; in sarcomas [11]; and in hematopoietic malignancies [12]. Serum Cu and Zn levels and their clinical usefulness in malignant states have been investigated, mainly in patients with hematological malignancies (e.g., leukemia and lymphoma) [13–15]. Low serum Zn and high Cu levels have been described in a few types of solid tumors (e.g., bronchogenic carcinoma, sarcomas, and carcinoma of digestive organs) [11, 16, 17], but little has been shown for malignant gynecological tumors. Some evidence suggested that Mn and Fe concentrations are higher in the serum of patients with malignant lymphoma and lung cancer [18, 19]. Midle et al. reported that Mn and Fe concentrations were significantly higher in colorectal cancer patients than in healthy subjects [20]. However, in patients with esophageal cancer and residents of areas with a high incidence of esophageal cancer, hair Mn content was significantly lower than in healthy subjects, and for residents in the low-incidence area, Mn content was also lower in rectal cancer tissue than in non-lesion tissue [21, 22].

Se is a well-known essential trace element that plays an important role as a constituent of the enzyme glutathione peroxidase [23]. This enzyme protects cellular components by reducing lipid hydroperoxides that may form as a result of the production of oxygen free radicals during aerobic metabolism [24]. Studies have shown that Se and Fe are essential trace elements and play an important role in malignant tumor incidence and progression. The relationships between dietary Se deficiency and cancer occurrence, serum and urinary Se-level variance, and cancer progression have been studied [25–27]. Selby and Friedman reported that a high content of body Fe stores could promote the development of cancer, at least for lung cancer [28]. Van Asperen et al. and Wurzelmann et al. found that increased Fe stores were associated with increased mortality or the incidence of cancer [29, 30]. At present, elements such as Cd, Ni and As are recognized as carcinogenic substances [31–34]. The levels of Cd, Ni and As in serum of patients with various diseases, including malignant tumors (breast cancer, lung cancer and prostate cancer), have been intensively investigated in recent years [35].

Uyghur women living in Xinjiang seem to suffer a higher disease burden of cervical cancer, in terms of both incidence and mortality, compared to women in other ethnic groups [36]. The low HPV infection rate is not in accordance with the current epidemiological status of Uyghur cervical cancer [37]. We assume factors other than HPV infection or components that may accelerate HPV infection and progression to cervical cancer may exist. In our former study, risk factors for HPV infection include sexual habit, marital status, and personal hygiene had been reported [38]. Xinjiang is a region located in northwestern China. The Uyghur people are distinctive from other ethnic groups in China in terms of customs and dietary habits, we assume that the environmental and nutritional factors may exist. Therefore, serum trace element levels and their relationship with HPV infection and cervical lesions need further exploration.

## Methods

### Patients and sample

In total, 5045 women were recruited for a cervical cancer screening survey in Maralbexi county, Xinjiang, between March 1, 2014 and June 15, 2014. careHPV, LBC (Liquid Based Cytology), VIA and VILI tests were adopted to screen for cervical cancer, and cervical biopsy by colposcopy was performed on women with any positive results. Some of the blood specimens were obtained from participants for trace element testing before the screening test began, while most were obtained with informed consent when the women went to the colposcopy visit. Blood specimens were collected from 646 women from the field survey, and 187 patients at the Affiliated Tumor Hospital of Xinjiang Medical University who were diagnosed with cervical lesions and came from Maralbexi county were also included in the survey. A total of 833 women were enrolled in the study. Inclusion criteria were age between 20 and 65 years, sexually active, no chemotherapy or radiotherapy, and no use of drugs or supplements containing trace elements. Written informed consent was obtained from each study participant before the study. The Ethical Committee of the Affiliated Tumor Hospital of Xinjiang Medical University approved the study.

### careHPV test

The new test, designated careHPV, is a signal-amplification assay that detects target HPV-DNA from 14 different carcinogenic HPV types (16, 18, 31, 33, 35, 39, 45, 51, 52, 56, 58, 59, 66, and 68). This is a qualitative test with the result of HPV positive or HPV negative.

### Assessment of serum trace element concentration

Blood samples were collecting with hemostix containing anticoagulant to avoid hemolyzis. The sample volume was 2–3 mL after centrifugation for all samples; serum was collected in disposable polypropylene tubes and kept frozen at − 70 °C until analyses were performed. Serum samples (0.5 mL) were digested by wet-washing using 5 mL of a nitric/perchloric acid mixture (4:1 *v*/v) in a 50-mL collection bottle and then placed on an electrical hot board at 120 °C until white smoke was produced. The samples were adjusted with deionized water to a final volume of 50 mL. The concentration of the trace elements Arsenic (As) and Selenium (Se) was determined by atomic absorption spectrophotometry. The concentrations of Cadmium (Cd), Nickel (Ni), Cuprum (Cu), Zinc (Zn), Manganese (Mn), and Iron (Fe) were determined by inductively coupled plasma atomic emission spectroscopy. Μmol/L is refers to μmol of trace element in per Liter of blood, while mg/k refers to mg of trace element in per kg of blood. The test was analyzed at the Xinjiang Uyghur Autonomous Region Research Institute of Analysis. All standard substances were obtained from the National Standard Material Center, Peking, China.

### Statistical analyses

All of the analyses were performed using SPSS version 19.0. Normal distribution and abnormal distribution of the data were tested by SPSS software by using Shapiro-Wilk test. Measurement data are expressed as the median (IQR) while enumeration data are expressed as the rate. Univariate analysis was performed with Chi-squared test between the HPV-positive and HPV-negative groups and between the case group and the control group. Multivariate analysis was performed with logistic regression. The significance level was set at *P* < 0.05.

## Results

The HPV infection rate in a total of 5045 women was 10.9%. The participants with HPV infection were regarded as the HPV-positive group (*n* = 551), and those without HPV infection formed the HPV-negative group (*n* = 282). Blood samples were obtained from most participants with HPV-positive results at the first screen when they went for the colposcopy visit, and women recruited from the hospital were also mostly infected with HPV. These reasons caused that in this study the HPV-positive rate was significantly higher compared to the total infection rate, which was as we mentioned above 10.9%. The participants with CIN ≥ 2 (cervical intraepithelial neoplasia grade 2+) (*n* = 150) were regarded as the case group, since patients who are CIN2+ need treatment by clinical procedure, and those characterized as CIN < 2 formed the control group (*n* = 683). The age range of women was 20–65 years, with a median age of 39.62 ± 9.58 years.

### Univariate analysis of trace elements and HPV infection

The median and interquartile range were adopted to present the results of serum trace element (Ni, Zn, Fe, Cu, Mn and Cd) tests as the measurement data were abnormally distributed. In the HPV-negative group, the serum levels of Ni, Zn, Fe, Cu, Mn, and Cd were 0.1821 mg/kg (*0.1678 mg/kg*), 105.9822 μmol/L (*44.7862* μ*mol/L*), 7.3485 mmol/L (*3.7108 mmol/L*), 19.8746 μmol/L (*19.8724* μ*mol/L*), 0.4879 μmol/L (*0.8125* μ*mol/L*), and 0.03618 μmol/L (*0.0334* μ*mol/L*), respectively, while those values were 0.1219 mg/kg (*0.1854 mg/kg*), 102.6875 μmol/L (*45.9654* μ*mol/L*), 6.5872 mmol/L (*3.1275 mmol/L*), 22.1089 μmol/L (*21.2617* μ*mol/L*), 0.5263 μmol/L (*0.8174* μ*mol/L*), and 0.0258 μmol/L (*0.0289* μ*mol/L*), respectively, in the HPV-positive group. To more accurately reflect the levels of the trace elements in serum and the relationship between HPV infection and Ni, Zn, Fe, Cu, Mn and Cd serum levels were classified according to the interquartile range of the HPV-negative group. The levels of serum Se and As were classified according to < 0.02 mg/kg and ≥ 0.02 mg/kg. Among women with an HPV infection, 42.47% had serum Se levels ≥0.02 mg/kg, and 60.44% had serum As levels ≥0.02 mg/kg. In the negative HPV group, 58.51% of women had Se levels ≥0.02 mg/kg, and 48.23% of women had As levels ≥0.02 mg/kg.

Results of the single factor analysis showed that the serum levels of Ni, Se, As and Cd were significantly different between the positive and negative HPV groups (*P <* 0.001; *P <* 0.001; *P* = 0.001; and *P* = 0.003). The serum levels of Zn, Fe, Mn and Cu in the samples with different HPV infection status were not significantly different (*P* = 0.087; *P* = 0.485; *P* = 0.145; and *P* = 0.251) (Table 1).

**Table 1:** Univariate analysis of trace elements and HPV infection in Uyghur women in China

Trace Elements	HPV (−) (*N* = 282)	HPV (+) (*N* = 551)		*P*
	n/%	n/%	χ^2^	
Ni (mg/kg)			56.905	< 0.001
Q1 (0.0212~)	70/24.82	282/51.18		
Q2 (0.1232~)	71/25.18	112/20.33		
Q3 (0.1892~)	70/24.82	81/14.70		
Q4 (0.2908~)	71/25.18	76/13.79		
Zn (μmol/L)			6.164	0.087
Q1 (28.4286~)	70/24.82	185/33.58		
Q2 (86.2117~)	71/25.18	120/21.78		
Q3 (106.5923~)	70/24.82	114/20.69		
Q4 (130.8978~)	71/25.18	132/23.95		
Fe (mmol/L)			2.449	0.485
Q1 (0.4357~)	70/24.82	137/24.86		
Q2 (5.5078~)	71/25.18	160/29.04		
Q3 (7.3654~)	70/24.82	114/20.69		
Q4 (9.1276~)	71/25.18	140/25.41		
Cu (μmol/L)			4.098	0.251
Q1 (2.0900~)	70/24.82	114/20.69		
Q2 (12.1317~)	71/25.18	125/22.69		
Q3 (20.0032~)	70/24.82	168/30.49		
Q4 (31.8041~)	71/25.18	144/26.13		
Mn (μmol/L)			5.396	0.145
Q1 (0.0100~)	70/24.82	109/19.78		
Q2 (0.1254~)	71/25.18	134/24.32		
Q3 (0.4947~)	70/24.82	175/31.76		
Q4 (0.9320~)	71/25.18	133/24.14		
Cd (μmol/L)			14.085	0.003
Q1 (0.0025~)	70/24.82	175/31.76		
Q2 (0.0248~)	71/25.18	109/19.78		
Q3 (0.0371~)	70/24.82	172/31.22		
Q4 (0.0571~)	71/25.18	95/17.24		
As (mg/kg)			11.301	0.001
<0.02	146/51.77	218/39.56		
≥ 0.02	136/48.23	333/60.44		
Se (mg/kg)			19.236	0.000
<0.02	117/41.49	317/57.53		
≥ 0.02	165/58.51	234/42.47		

### Logistic regression analysis of the relation between trace elements and HPV infection

Serum levels of Ni, Zn, Cd, As and Se, which were shown as statistically significant in the univariate analyses, were also analyzed by the logistic regression model, with adjustments for age and education level. The concentration of As ≥0.02 mg/kg was a risk factor for HPV infection in Uyghur women (OR = 1.664, 95% CI: 0.989–2.800; (*P* = 0.025), while the concentrations of Ni ≥0.1232 mg/kg (OR = 0.395, 95% CI: 0.194–0.802; (*P* = 0.004) and Se ≥0.02 mg/kg (OR = 0.499, 95% CI: 0.297–0.839; (*P* = 0.010) were protective factors (Table 2).

**Table 2:** Multivariate Analysis of Trace Elements and HPV Infectionin Uyghur women in China

Trace Elements	RC	SE	Wald *χ*^*2*^	OR	95% CI	*P*
Ni (mg/kg)			25.694			0.000
Q2 (0.1232~)	−0.382	0.274	8.665	0.395	0.194–0.802	0.004
Q3 (0.1892~)	−0.419	0.338	9.028	0.347	0.169–0.714	0.001
Q4 (0.2908~)	−0.457	0.365	20.867	0.248	0.115–0.536	<0.001
Se (mg/kg) ≥0.02	−0.356	0.262	7.904	0.499	0.297–0.839	0.010
As (mg/kg) ≥0.02	0.283	0.196	6.379	1.664	0.989–2.800	0.025

### Univariate analysis of trace elements and CIN2+ in Uyghur women

The median and interquartile range of serum levels in the control group were as follows: Ni: 0.0942 mg/kg (0.1047 mg/kg), Zn: 93.6937 μmol/L (48.9736 μmol/L), Fe: 7.2894 mmol/L (3.0267 mmol/L), Cu: 24.1875 μmol/L (22.1672 μmol/L), Mn: 0.4984 μmol/L (0.7098 μmol/L), and Cd: 0.0418 μmol/L (0.02876 μmol/L. In the case group, the corresponding values were as follows: Ni: 0.1584 mg/kg (0.1406 mg/kg), Zn: 105.5670 μmol/L (49.7065 μmol/L), Fe: 6.9153 mmol/L (3.8024 mmol/L), Cu: 21.8746 μmol/L (19.6439 μmol/L), Mn: 0.5493 μmol/L (0.7303 μmol/L), and Cd: 0.0359 μmol/L (0.0319 μmol/L). To more accurately reflect the levels of the trace elements in serum and the relationship with CIN2+, the serum levels of Ni, Zn, Fe, Cu, Mn and Cd were classified according to the interquartile range of the control group. The levels of serum As and Se were classified according to < 0.02 mg/kg and ≥ 0.02 mg/kg. In total, 81.33% of women in the case group had serum As levels ≥0.02 mg/kg, and 15.33% had serum Se levels ≥0.02 mg/kg. In the control group, 50.07% of women had As levels ≥0.02 mg/kg, and 55.78% had Se levels ≥0.02 mg/kg.

Results of the single factor analysis showed that the serum levels of Fe and As tended to increase in the case group (*P <* 0.001), while the levels of Ni and Se had a downward trend (*P <* 0.001). The serum levels of Zn, Cd, Mn and Cu in samples from different groups were not significantly different (Table 3).

**Table 3:** Univariate analysis of trace elements and CIN2+ in Uyghur women

Trace Element	CIN < 2(*N* = 683)	CIN2+ (*N* = 150)	*P*
n (%)	n (%)	χ^2^	
Ni (mg/kg)		47.795	<0.001
Q1 (0.0212~)	170/24.89	78/52.00	
Q2 (0.0965~)	171/25.04	32/21.33	
Q3 (0.1584~)	171/25.04	14/9.33	
Q4 (0.2364~)	171/25.04	26/17.33	
Zn (μmol/L)		7.137	0.068
Q1 (26.4286~)	170/24.89	51/34.00	
Q2 (80.7867~)	171/25.04	39/26.00	
Q3 (105.5670~)	171/25.04	21/14.00	
Q4 (130.4932~)	171/25.04	39/26.00	
Fe (mmol/L)		30.411	<0.001
Q1 (0.4357~)	170/24.89	12/8.00	
Q2 (5.1895~)	171/25.04	28/18.67	
Q3 (6.9153~)	171/25.04	55/36.67	
Q4 (8.9919~)	171/25.04	55/36.67	
Cu (μmol/L)		4.682	0.328
Q1 (2.0900~)	170/24.89	21/14.00	
Q2 (12.2423~)	171/25.04	35/23.33	
Q3 (21.3933~)	171/25.04	55/36.67	
Q4 (31.8862~)	171/25.04	39/26.00	
Mn (μmol/L)		5.579	0.134
Q1 (0.0100~)	64/24.8	48/32.00	
Q2 (0.2093~)	65/25.2	32/21.33	
Q3 (0.5493~)	65/25.2	42/28.00	
Q4 (0.9396~)	64/24.8	28/18.67	
Cd (μmol/L)		7.040	0.071
Q1 (0.0025~)	170/24.89	42/28.00	
Q2 (0.0224~)	171/25.04	28/18.67	
Q3 (0.0359~)	171/25.04	50/33.33	
Q4 (0.0543~)	171/25.04	30/20.0	
As (mg/kg)		48.707	<0.001
<0.02	341/49.93	28/18.67	
≥ 0.02	342/50.07	122/81.33	
Se (mg/kg)		80.567	<0.001
<0.02	302/44.22	127/84.67	
≥ 0.02	381/55.78	23/15.33	

### Logistic regression analysis of the relation between trace elements and CIN2+ in Uyghur women

Serum levels of Ni, Fe, As and Se, which were shown as statistically significant in the univariate analyses, were analyzed by the logistic regression model, and age and education level were adjusted. Concentrations of As ≥0.02 mg/kg (OR = 4.466, 95% CI: 2.218–8.996; *P* < 0.001) and Fe ≥6.9153 mmol/L (OR = 5.974, 95% CI: 2.029–17.586; *P* = 0.003) were risk factors for CIN2+ in Uyghur women, while concentrations of Ni ≥0.0965 mg/kg (OR = 0.371, 95% CI: 0.175–0.788; *P* = 0.011) and Se ≥0.02 mg/kg (OR = 0.124, 95% CI: 0.058–0.267; *P* < 0.001) were protective factors (Table 4).

**Table 4:** Multivariate analysis of trace elements and CIN2+ in Uyghur Women

Trace Elements	RC	SE	Wald *χ*^*2*^	OR	95% CI	*P*
Ni (mg/kg)			19.526			<0.001
Q2 (0.0965~)	−0.347	0.252	9.854	0.371	0.175–0.788	0.011
Q3 (0.1584~)	−0.513	0.364	14.835	0.144	0.054–0.381	<0.001
Q4 (0.2364~)	−0.449	0.341	12.458	0.293	0.127–0.678	<0.001
Fe (mmol/L)			10.683			0.002
Q2 (5.1895~)	0.359	0.272	5.843	2.809	0.873–9.034	0.088
Q3 (6.9153~)	0.404	0.287	10.596	5.974	2.029–17.586	0.003
Q4 (8.9919~)	0.381	0.263	10.473	5.416	1.839–15.948	0.004
As (mg/kg) ≥0.02	0.492	0.281	13.837	4.466	2.218–8.996	<0.001
Se (mg/kg) ≥0.02	−0.557	0.379	15.074	0.124	0.058,0.267	<0.001

## Discussion

Studies suggest that the content of trace elements has a great influence in the body on cellular structure stability, the stability of nucleic acids and immunity [39]. Our data indicate that Se concentration ≥ 0.02 mg/kg was a protective factor for HPV infection and high-grade cervical lesions, including cervical cancer. This finding was in agreement with the findings of Psathakis et al. [40]. Se deficiency may be caused by low Se content in food, which mostly contributes to the intake of Se by humans. The results of the study in Qi Dong County of China showed that the incidence of hepatitis B virus infection and primary liver cancer was significantly decreased by supplemental Se in the population’s diet. The anticancer effect of Se has been supported by animal experiments, human epidemiological investigations, and intervention trials [28, 41]. The biological effects of Se play an important role in the human immune system, proliferation of B-lymphocytes, and enhanced T-cell function, and Se is an essential component of antioxidants, such as the enzymes glutathione peroxidase and thioredoxin reductase [42]. Antioxidants are effective in controlling intracellular peroxide levels in mitochondria, and the cytoplasm protect cells from oxidative damage from reactive oxygen species released to kill engulfed bacteria [43]. However, the mechanism of Se as an anticancer agent is not clear yet; further research, especially on individual selenoproteins, is needed to understand the function of selenium in each of the cell types of the immune system.

Our results were not consistent with the results reported by Ji et al. and Wu et al. [42, 44], which suggested that the high level of Ni was a high risk factor for causing nasopharyngeal carcinoma, lung cancer and colorectal cancer. In our data, serum Ni content (≥0.0965 mg/kg) was significantly decreased in cervical cancer patients versus healthy subjects; Ni concentration ≥ 0.1232 mg/kg was a protective factor for HPV infection, which may be influenced by multiple factors. However, further research is needed to clarify the underlying mechanisms and the biological roles of trace elements. Our results showed that the elevated serum levels of As (≥ 0.02 mg/kg) might be a risk factor for HPV infection, cervical high-grade lesions and cervical cancer. Changing cervical epithelial cell differentiation and proliferation could induce the occurrence of cervical lesions [45]. However, the pathomechanism of plasma with alterations remains unclear; further investigation is needed to determine whether As plays a role in cervical cancer incidence and HPV infection. Fe can participate in the proliferation of tumor cells, with DNA oxidative damage leading to the occurrence of tumors. Multivariate analysis showed that elevated serum levels of Fe (≥ 6.9153 mmol/L) might be a risk factor for cervical high-grade lesions and cervical cancer. This finding is in agreement with the results of Zhang CG et al. [46].

Zn plays an important role in cell division, growth, DNA synthesis, RNA transcription, and aspects of the immune system [47]. Zn is an antioxidant or free-radical scavenger. Zn deficiency may be the cause of malignant tumor occurrence. Animal models have demonstrated that Zn deficiency is involved in several stages of malignant transformation, initiation, and promotion [48]. In a recent study, serum Cu levels were implicated in patients with ovarian, esophageal, lung, and colorectal cancer and hematological neoplastic diseases [21, 24, 25, 49, 50]. The pathomechanism of Cu levels in plasma and tissue elevation remains unclear. However, Machacek et al. concluded that changes in the activity of the enzymes sialotransferase and neuraminidase led to increased levels of plasma Cu; reduced catabolism of protein-carrying ceruplasmin also caused an elevation in Cu levels [51]. Reports showed that the serum levels of Mn are significantly higher in cervical cancer and colorectal cancer patients than in healthy subjects [22, 42]. Mn is an essential trace element in the body. Mn has several chemical and biochemical properties similar to iron, and there is evidence of metabolic interactions between the two metals, especially at the level of intestinal absorption [52]. The Cd carcinogenic mechanism mainly includes abnormal expression of DNA, induced oxidative stress, inhibition of cell DNA repair, and inhibition of apoptosis.

In our data, the levels of Mn, Cu, Cd and Zn were slightly different in the two groups, but the difference was not statistically significant. The exact relationship between these elements and HPV infection and CIN2+ in Uyghur women needs to be verified in further studies. Although the findings from this epidemiological study cannot establish causality, they provide a solid base that low concentrations of Se and Ni and the high concentration of As might be related to HPV infection and CIN2+ in Uyghur women in rural China. Well-designed cohort studies or clinical trials may be warranted to confirm these associations.

## Conclusion

Based on our study, we conclude that low serum concentrations of Se and Ni and a high serum concentration of As might be related to HPV infection and CIN2+ in Uyghur women in rural China, while the serum levels of Cd, Zn, Fe, Mn and Cu were no obvious influence on HPV infection and cervical lesion of Uyghur women. The result will provide a theoretical basis for our future nutritional intervention in cervical cancer prevention project.

## Abbreviations

As: Arsenic
Cd: Cadmium
CI: Confidence Interval
CIN: Cervical Lesion in Neoplasia
Cu: Cuprum
Fe: Iron
HPV: Human Pappilloma Virus
IQR: Interquartile Range
LBC: Liquid Based Cytology
Mn: Manganese
Ni: Nickle
OR: Odd Ratio
Se: Selenium
SPSS: Statistic Package for Social Science
VIA: Visual Inspeciton with Actic Acid
VILI: Visual Inspection with Lugol Iodine
Zn: Zinc

## References

[1] GLOBOCAN. Estimated cancer incidence, mortality and prevalence worldwide in 2012. 2012. [2015–02-02]. http://gco.iarc.fr/.

[2] Walboomers JM, Jacobs MV, Manos MM, Bosch FX, Kummer JA, Shah KV, Snijders PJ, Peto J, Meijer CJ, Munoz N (1999). Human papillomavirus is a necessary cause of invasive cervical cancer worldwide. J Pathol.

[3] Crum CP, Abbott DW, Quade BJ (2003). Cervical cancer screening: from the papanicolaou smear to the vaccine era. J Clin Oncol.

[4] Lv Y, Han L, Yuan C, Guo J (2009). Comparison of hypoglycemic activity of trace elements absorbed in fermented mushroom of Coprinus comatus. Biol Trace Elem Res.

[5] Xu Q, Guo J (2009). Activity and toxicity of Cr (III)-enriched Grifola frondosa in insulin-resistant mice. Biol Trace ElemRes.

[6] Keiderling W, Scharpf H (1954). Uber die klinische Bedeutung der Serum Kupfer und Serum Eisenbestimmung bei neoplastichen Krankheitszustanden. Munch Med Wochenschr.

[7] De Jorge FB, Goes JS, Guedes JL, De Ulhoa Cintra AB (1965). Biochemical studies on copper, copper oxidase, magnesium, sulfur, calcium and phosphorus in cancer of the breast. Clin Chim Acta.

[8] O’Leary JA, Feldman M (1970). Serum copper alterations in genital cancer. Surg Forum.

[9] Albert L, Hienzsch E, Arndt J, Kriester A (1972). Bedeutung und Veriinderungen des serum-Kupferspiegels warend und nach der Bestrahlung von Harnblasenkarzinomen. [significance and changes of serum copper level during and following irradiation for bladder carcinoma]. Z Urol Nephrol.

[10] Kolarić K, Roguljić A, Fuss V (1975). Serum copper levels in patients with solid tumors. Tumori.

[11] Fisher GL, Byers VS, Shifrine M, Levin AS (1976). Copper and zinc levels in serum from human patients with sarcomas. Cancer.

[12] Delves HT, Alexander FW, Lay H (1973). Copper and zinc concentration in the plasma of leukaemic children. Br J Haematol.

[13] Alexander FW, Delves HT, Lay H (1972). Plasma copper and zinc in acute leukaemia. Arch Dis Child.

[14] Hrgovcic M, Tessmer CF, Thomas FB, Ong PS, Gamble JF, Shullenberger CC (1973). Serum copper observations in patients with malignant lymphoma. Cancer.

[15] Thorling EB, Thorling K (1976). The clinical usefulness of serum copper determinations in Hodgkin’s disease. A retrospective study of 241 patients from 1963-1973. Cancer.

[16] Inutsuka S, Araki S (1978). Plasma copper and zinc levels in patients with malignant tumors of digestive organs: clinical evaluation of the C1/Zn ratio. Cancer.

[17] Strain WH, Mansour EG, Flynn A, Pories WJ, Tomaro AJ, Hill OA (1972). Plasma-zinc concentration in patients with bronchogenic cancer (letter). Lancet.

[18] Zhao X, Han C, Jing J (1998). Relationship of serum trace elements to lung cancer and its clinical application. Zhonghua Liu Xing Bing Xue Za Zhi.

[19] Ho C-m J, Zheng S, Comhair SA, Farver C, Erzurum SC (2001). Differential expression of manganese superoxide dismutase and catalase in lung cancer. Cancer Res.

[20] Milde D, Novák O, Stu ka V, Vyslou il K, Machá ek J (2001). Serum levels of selenium, manganese, copper and iron in colorectal cancer patients. Biol Trace Elem Res.

[21] Han CZ, Jing JX (1995). Relationship between trace elements in hair and esophageal cancer. Acta Nutr Sin.

[22] Han C, Liang X, Jing J (1999). Study on the association and significance between trace elements and rectal cancer. Zhonghua Liu Xing Bing Xue Za Zhi..

[23] Simonoff M, Simonoff G (1991). Le sélénium et la vie.

[24] Schrauzer GN (1992). Selenium. Mechanistic aspects of anticarcinogenic action. Biol Trace Elem Res.

[25] Li WG, Huang QS, Yu SY (1993). Six-year prospective observation on ingesting selenium-salt to prevent primary liver cancer. Chin J Cancer.

[26] Zhang YJ, Jing JX, Han CZ (2002). Impairment of antioxidant system in human esophageal cancer. Chin J Exp Surg.

[27] Navarrete M, Gaudry A, Revel G, Martínez T, Cabrera L (2001). Urinary selenium excretion in patients with cervical uterine cancer. BTER.

[28] Selby JV, Friedman GD (1988). Epidemiologic evidence of an association between body iron stores and risk of cancer. Int J Cancer.

[29] van Asperen IA, Feskens EJ, Bowles CH, Kromhout D (1995). Body iron stores and mortality due to cancer and ischaemic heart disease: a 17-year follow-up study of elderly men and women. Int J Epidemiol.

[30] Wurzelmann JI, Silver A, Schreinemachers DM, Sandler RS, Everson RB (1996). Iron intake and the risk of colorectal cancer. Cancer Epidemiol Biomark Prev.

[31] Yu X, Filardo EJ, Shaikh ZA (2010). The membrane estrogen receptor GPR30 mediates cadmium-induced proliferation of breast cancer cells. Toxicol Appl Pharmacol.

[32] Tokumoto M, Fujiwara Y, Shimada A, Hasegawa T, Seko Y, Nagase H (2011). Cadmium toxicity is caused by accumulation of p53 through the down-regulation of Ube2d family genes in vitro and in vivo. J Toxicol Sci.

[33] Hartwig A (2013). Cadmium and cancer. Met Ions Life Sci.

[34] Wong VC, Morse JL, Zhitkovich A (2013). p53 activation by Ni (II) is a HIF-1α independent response causing caspases 9/3-mediated apoptosis in human lung cells. Toxicol Appl Pharmacol.

[35] Strumylaite L, Bogusevicius A, Abdrachmanovas O, Baranauskiene D, Kregzdyte R, Pranys D (2011). Cadmium concentration in biological media of breast cancer patients. Breast Cancer Res Treat.

[36] Li J, Kang LN, Qiao YL (2011). Review of the cervical cancer disease burden in mainland China. Asian Pac J Cancer Prev.

[37] Ablimit T, Abuduxkur G (2015). Analysis of cervical cancer screening in Xinjiang Hotan Uyghur women. J Xinjiang. Med Univ.

[38] Abulizi G, Li H, Mijiti P, Abulimiti T, Cai J, Gao J, Meng D, Abula R, Abudereyimu T, Aizezi A, Qiao YL (2017). Risk factors for human papillomavirus infection prevalent among Uyghur women from Xinjiang, China. Oncotarget.

[39] Thompson FE, Patterson BH, Weinstein SJ, McAdams M, Spate VL, Hamman RF (2002). Serum selenium and the risk of cervical cancer among women in the United States. Cancer Causes Contr.

[40] Psathakis D, Wedemeyer N, Oevermann E, Krug F, Siegers CP, Bruch HP (1998). Blood selenium and glutathione peroxidase status in patients with colorectal cancer. Dis Colon Rectum.

[41] Singh V, Garg AN (1998). Trace element correlations in the blood of Indian women with breast cancer. Biol Trace Elem Res.

[42] Ji WD, Chen JK, Lu JC, Wu ZL, Yi F, Feng SM (2006). Alterations of FHIT gene and P16 gene in nickel transformed human bronchial epithelial cells. Biomed Environ Sci.

[43] Medina D, Lane HW, Tracey CM (1983). Selenium and mouse tumorgenesis: an investigation of possible mechanisms. Cancer Res (Suppl).

[44] Wu CH, Tang SC, Wang PH, Lee H, Ko JL (2012). Nickel-induced epithelial-mesenchymal transition by reactive oxygen species generation and E-cadherin promoter hypermethylation. J Biol Chem.

[45] Lu G, Xu H, Chang D, Wu Z, Yao X, Zhang S (2014). Arsenic exposure is associated with DNA hypermethylation of the tumor suppressor gene p16. J Occup Med Toxicol.

[46] Zhang CG, Zhang F (2015). Iron homeostasis and tumorigenesis: molecular mechanisms and therapeutic opportunities. Protein Cell.

[47] Prasad AS (1998). Zinc in human health: an update. J Trace Elem Exp Med.

[48] Newberne PM, Schrager TF, Broitman S (1997). Esophageal carcinogenesis in the rat: zinc deficiency and alcohol effects on tumor induction. Pathobiology.

[49] Cheng Y, Han CZ, Jing JX (1997). The correlation of trace elements with ovarian cancer and clinical application value. J Shanxi Med.

[50] Cunzhi H, Jiexian J, Xianwen Z, Jingang G, Suling H (2001). Classification and prognostic value of serum copper/zinc ratio in Hodgkin’s disease. Biol Trace Elem Res.

[51] Machacek J, Mensik P, Bicik V (1984). Serum levels of copper and zinc in breast cancer and its possible using in clinical practice. New Clin Oncologist.

[52] Chua AC, Morgan EH (1996). Effects of iron deficiency and iron overload on manganese uptake and deposition in the brain and other organs of the rat. Biol Trace Elem Res.

